# High frequency of *Candida krusei* colonization in critically ill pediatrics: A cross-sectional study in children’s medical center, Tehran, Iran

**DOI:** 10.18502/cmm.8.2.10329

**Published:** 2022-06

**Authors:** Amirhossein Davari, Jalal Jafarzadeh, Mohammad Taghi Hedayati, Tahereh Shokohi, Mahdi Abastabar, Bahram Nikmanesh, Maryam Moazeni

**Affiliations:** 1 Student Research Committee, Mazandaran University of Medical Sciences, Sari, Iran; 2 Invasive Fungi Research Center, Communicable Diseases Institute, Mazandaran University of Medical Sciences, Sari, Iran; 3 Department of Medical Mycology and Parasitology, School of Medicine, Babol University of Medical Sciences, Babol, Iran; 4 Department of Medical Mycology, School of Medicine, Mazandaran University of Medical Sciences, Sari, Iran; 5 Department of Medical Laboratory Sciences, School of Allied Medical Sciences, Tehran University of Medical Sciences, Tehran, Iran

**Keywords:** *Candida* Colonization index, Candidiasis, ICU, Pediatric, PICU

## Abstract

**Background and Purpose:**

This study aimed to evaluate the species distribution and susceptibility pattern of the strains isolated from Candida colonization in pediatric patients staying at pediatric intensive care unit (ICU) and infant ICU of Children’s Medical Center in Tehran, Iran.

**Materials and Methods:**

This study was conducted in the Children’s Medical Center in Tehran, Iran. In total, 440 samples from 56 patients with oral cavity, skin surrounded catheters, and ear, throat, nasal, and urine cultures were collected. All patients were evaluated in terms of *Candida* colonization on the admission day as well as the days 7, 14, and 28 according to the previous studies. CHROMagar *Candida* medium was applied for primary/multiple species identification and the isolates were identified by using polymerase chain reaction-based methods to the species-specific complex level. The antifungal susceptibility test was performed according to the Clinical and Laboratory Standards protocol published as M27-A3 and M60 documents.

**Results:**

In total, 136 yeast samples from 26 individuals (30.9%) out of 440 samples were considered colonization. The most prevalent species in IICU was *C. albicans* (27%, n=20) followed by *C. krusei* (24 %, n=18) and *C. parapsilosis* (16%, n=12). In PICU, the predominant species was *C. krusei* (40%, n=24) followed by *C. parapsilosis* (18%, n=11) and *C. dubliniensis* (16%, n=10). Among the 40 tested isolates from both units, fluconazole-resistant isolates (n=11, 8.15%) were determined according to the new breakpoints. In the case of echinocandins, 2 isolates, including C. albicans (n=1) and C. krusei (n=1) were resistant against both caspofungin and anidulafungin (totally 1.48%).

**Conclusion:**

In the present study, since *C. krusei* is intrinsically-resistance against fluconazole, emphasizing the importance of species-level identification of *Candida* isolates is outstanding. However, according to the antifungal susceptibility testing results, only 7.2% of the strains were resistant to fluconazole. It would be beneficial to monitor the ICU patients who are at high risk of invasive *Candida* infection.

## Introduction

Infections caused by *Candida* species, known as invasive *Candida* infection, are an important cause of morbidity and mortality among hospitalized children, especially in critically ill children and neonates. Among children, *Candida* infections are considered the third most common cause of healthcare-associated bloodstream diseases and the second most usual etiologic agent of central catheter-associated bloodstream infections. [ 1 ]. Recently, an increasing tendency has been reported in the incidence rate of non-albicans *Candida* (NAC) species in pediatric intensive care units (PICUs) [ 2 ]. 

A remarkable difference has been revealed in the rate of candidemia in developing countries, compared to the developed countries (42.7 vs. 0.043–0.47 per 1000 admissions) [ 3 ]. Accordingly, in the developing countries, non-*albicans* species receive considerable attention [ 4 - 7 ]. Early empirical management of invasive candidiasis has pros and cons. Apart from survival improvement, misuse and overuse of antifungals result in an increase in the financial fee and also the emergence of antifungal-resistant strains as the causative agent of infections [ 8 ]. 

The first stage of severe infections, such as candidemia, is *Candida* colonization. Colonization can be described as the presence of the yeast with growth and multiplication, but without any obvious clinical manifestations or detectable immune response in the host at the time of isolation [ 9 ]. Fungal colonization has been considered and documented as a risk factor that acts independently in the elucidation of invasive candidiasis [ 10 ]. 

The colonization status is believed to be potentially predictive of the possibility of invasive candidiasis. This has been observed in many studies that have included *Candida* colonization as part of a scoring system and a prediction tool for the development of invasive candidiasis [ 11 - 13 ]. The Prevalence of *Candida* colonization in critically ill pediatric patients has been reported in up to 60% of the cases after 4-7 days in the major care unit [ 14 - 17 ]. In the present study, the distribution of *Candida* species, which colonized the children hospitalized at ICU units (PICU and IICU) in Tehran Children Center, has been studied.

## Materials and Methods

### Patients and sampling

This cross-sectional study was conducted in IICU (infant intensive care unit) and PICU sections of the Children Medical Center in Tehran between March 2019 and September 2019. In total, 440 samples were collected from 55 patients who were within the age range of 1 day to 21 years. Due to the chance of the colonization of patients with Candida strains, those who were supposed to stay in these units for > 3 days were included; however, the patients who stayed for < 7 days were excluded from the study [ 18 ]. Signed informed consent was obtained from parents before sampling. 

Regarding all patients, the data sheets were used to record age, gender, diagnosis, underlying diseases, laboratory findings at the time of admission and during follow-up, and length of ICU stay. According to a previous study [ 19 ], body sites more prone to *Candida* colonization include the oral cavity and the skin surrounding catheters. Accordingly, ear, throat, nasal swabs, and urine cultures were performed at the time of admission and every week throughout their stay. Swab samples were obtained by using swab sticks while urine samples were collected as mid-stream urine or from a urinary catheter. 

*Candida* Colonization was evaluated for each patient on the admission day as well as the days 7, 14, and 28 according to the previous studies which defined colonization with a colonization index higher than 2 (CI>2) [ 20 ]. All the samples taken by the swab were transferred to 1 ml of normal saline containing 1% Tween 80. Afterward, 100 µL of the suspension was cultured on Sabouraud dextrose agar (HIMEDIA, India) containing chloramphenicol as well as chromogenic medium (CHROMagar *Candida*, CHROMagar Microbiology, France) for primary detection. The study was approved by the Ethics Committee of Mazandaran University of Medical Sciences (Code IR.MAZUMS.REC .1399.7165).

### Identification of the isolates

Primary as well as multiple-species identification was performed based on the colony color grown on CHROMagar *Candida* culture medium. Genomic DNA was extracted from all the recovered yeast isolates according to the previously described method [ 21 ]. Molecular identification of common *Candida* species was carried out according to an already described polymerase chain reaction-restriction fragment length polymorphism (PCR-RFLP) method [ 22 ]. The amplifications were conducted according to the following instruction: an initial denaturation at 95 °C for 5 min followed by 35 cycles of denaturation at 95 °C for 15 s, annealing at 56 °C for 30 s, and elongation at 72 °C for 30 s. 

After the final cycle, the samples were incubated for 5 min at 72 °C. The PCR products were digested with 5 U of the restriction enzyme *MspI* (Fermentas, Vilnius, Lithuania) following gel electrophoresis on 2% agarose gel. *Candida albicans* and *Candida parapsilosis* species complexes were distinguished from each other by the analysis of the hyphal wall protein and intein-containing vacuolar ATPase precursor genes, respectively, based on previously described protocols [ 23 , 24 ] (Table 1). 

**Table 1:** Primer sequences for species identification of Candida isolates.

Name	Primer’s sequences (5’ → 3’)	References
PCR-RFLP for all isolates		
ITS1-F	GCACCTTCAGTCGTAGAGACG	[ 22 ]
ITS4-R	GCACCTTCAGTCGTAGAGACG	
*Candida albicans* species complex		
HWP-F	GCTACCACTTCAGAATCATCATC	[ 23 ]
HWP-R	GCACCTTCAGTCGTAGAGACG	
*Candida parapsilosis* species complex		
GAGAAAGCACGCCTCTTTGC	OM-F	[ 24 ]
OM-R	TCAGCATTTTGGGCTCTTGC	

### Antifungal Susceptibility Testing

Guidelines of the Clinical and Laboratory Standards, M27-A3 and M60 [ 25 , 26 ], were applied for performing antifungal susceptibility testing (AFST). Fluconazole (FLZ), itraconazole (ITZ), voriconazole (VRZ), ravuconazole (RVZ), and isavuconazole (ISZ) were used as azole antifungals. Caspofungin (CAS) and anidulafungin (AFG) were applied as echinocandins and amphotericin B was employed as polyene antifungals. 

All antifungal agents were provided by Sigma-Aldrich (Germany) and dissolved in dimethyl sulfoxide. Afterward, they were diluted in a standard RPMI 1640 medium (Sigma Chemical Co.) which was previously buffered to pH 7.0 with the use of 0.165 3-(N-Morpholino) propane sulfonic acid (sigma chemical Co.) to yield their two-fold initial concentrations. Subsequently, they were distributed into 96-well microdilution trays (Nunc, UK) with a final concentration of 0.016-16μg/ml for all, except FLZ. 

Regarding FLZ, this concentration was considered 0.063-64 μg/ml. Fresh yeast suspensions were prepared and adjusted by spectrophotometric measurements at 530 nm wavelengths to a percent transmittance range of 75-77. A working suspension of 2.5-5×10^3^ colony-forming unit/ml was applied. An equal volume of antifungal agents, as well as yeast suspension, were added to each well. Drug-free and yeast-free wells were included as positive and negative controls, respectively. The microdilution plates were incubated at 35 °C and examined visually after 24 h. Candida krusei (ATCC6258) and *C. parapsilosis* (ATCC 22019) were used as quality controls

### Statistical analysis

The data were recorded using Microsoft Excel 2007 (Microsoft Corp, Redmond, WA, USA) and analyzed using SPSS software (version 16; SPSS Inc., Chicago, IL, USA). Quantitative variables were compared by using the Mann-Whitney U test. Categorical variables were compared using categorical variables, and the Fisher exact test was used to establish differences in their distributions between the subgroups. *P-values* less than 0.05 were considered statistically significant.

## Results

### Patients and sampling

In total, 440 samples were obtained from 55 patients (children/infants/newborns) in IICU and PICU. More precisely, 28 patients from IICU and 27 from the PICU unit were enrolled in the study, including 18 females and 37 males. The mean ages of the subjects were 5.9±3.1 and 3.05± 2.7 years in patients with and without yeast growth, respectively. No significant difference was found between positive and negative-grown yeast groups in terms of age (*P*>0.05). Out of 440 samples, 135 samples from 27 individuals (30.68 %) were considered positive cultures with at least one colony yeast growth. No significant difference was observed regarding gender (*P*>0.05). Moreover, no significant differences were observed regarding underlying diseases (*P*>0.05). Yeast growth was mostly reported in oral cavity (n=53, 38.7%) as well as aural (n=32, 23.3%) and nasal (n= 26, 23.3%) samples. Table 2 indicates the demographic data for the patients with grown isolated strains.

**Table 2:** Demographic data of patients and distribution of the yeast species stayed at IICU and PICU.

	Underlying Disease	Gender	Age (year)	Identified Species
**1**	COPD*	M	2	*C. parapsilosis (2) C. albicans (2). C. dubliniensis (2) .C. krusei (2 )*
**2**	Asthma	M	1	*C. krusei (1). C. guilliermondii (3)*
**3**	ALL	F	5	*C. krusei (3).C.albicans (4). C.gilermondi ( 1)*
**4**	ALL	F	7	*C. albicans (5). C. parapsilosis (2 )*
**5**	VSD**	F	3m	*C. parapsilosis (2)*
**6**	Laryngomalacia	M	2m	*C. africana (2)*
**7**	ALL	M	10	*C. krusei (8).C.albicans (3). C. parapsilosis (2).C.lusitania (4)*
**8**	Distress Breathing	F	6m	*C. albicans (3). C. krusei (2)*
**9**	ALL	F	3m	*C. albicans (4).C. galbrata (3)*
**10**	Distress Breathing	F	10	*C. krusei (2)*
**11**	ALL	M	5	*C. krusei (4).C.albicans (2). C. dubliensis(1)*
**12**	Seizure	M	8m	*C. krusei (2)*
**13**	Pneumonia	F	4m	*C. parapsilosis (1). C. dubliniensis (1)*
**14**	Meningitis	M	11	*C. glabrata (2)*
**15**	Asthma	F	4m	*C .glabrata (2)*
**16**	ALL***	F	9m	*C. dubliniensis (4)*
**17**	Bronchitis	M	3m	*C. krusei (9).C. parapsilosis (1) C. glabrata (1)*
**18**	ALL	M	14	*C. albicans (3) C .dubliniensisn (2) C. glabrata (3) C. krusei (1) C. parapsilosis(1)*
**19**	Down Syndrome	M	9 m	*C. krusei (1) C. parapsilosis (1)*.
**20**	Thalassemia	M	12m	*C. parapsilosis (2) C. krusei (1). C. dubliniensis (1) C .albicans(1)*.
**21**	CVID^£^	M	2	*C. parapsilosis (2) C.albicans(1) C. krusei (1)*.
**22**	ALL	M	4	*C. krusei (1), C. parapsilosis (3)*
**23**	Distress Breathing	F	8m	*C. krusei (2), C. parapsilosis(1)*
**24**	Meningitis	M	3	*C. glabrata (2), C. guilliermondii (1)*
**25**	Asthma	M	11m	*C. parapsilosis (1), C. glabrata (1)*
**26**	ALL	F	2	*C. parapsilosis (1), C. glabrata (1), C. kefyr (1)*
**27**	Bronchitis	F	5	*C. parapsilosis (1), C. glabrata*

### Identification of the isolates

Notably, *C. krusei* was the most prevalent species in PICU and the second in IICU. Out of 42 isolated *C. krusei* strains, nasal (n=15), aural (n=12), oral (n=9), and urine (n=6) were the most prevalent sites colonized with the species. Moreover, *C. krusei* was not isolated from the throat and skin. Regarding the distribution of the species in IICU, *C. albicans*, *C. krusei*, *C. parapsilosis*, *C. dubliniensis*, *C. glabrata*, *C. guilliermondii*, *C. lusitaniae*, *C. Africana*, and *C. kefyr* were detected in 27% (n=20), 24% (n=18), 16% (n=12), 10% (n=7), 7% (n=5), 7% (n=5), 5% (n=4), 3% (n=2), and 1% (n=1) of the subjects. 

**Figure 1:**
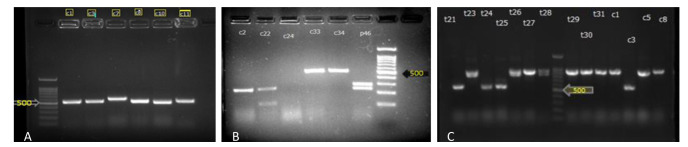
(A): Results of gel electrophoresis of PCR products of the ITS region. (B): Electrophoresis image of PCR-RFLP (ITS region digested with Msp1) products on 2% agarose gel [M: 100 bp DNA marker; C2: *C. krusei*, C22: *C. lusitania*, C33, C34: *C. parapsilosis*, P46: *C. albicans*. (C) Results of gel electrophoresis of PCR products of the HWP region; T23, T26-31, C1, C5, and C8: *C. albicans*, T24, T25, and C3: *C. dubliniensis*

In PICU, the distribution of Candida species was as follow: *C. krusei* 40% (n=24), *C. parapsilosis* 18% (n=11), *C. dubliniensis* 16% (n=10), *C. albicans* 13% (n=8), and *C. glabrata* 13% (n=8). Overall, *C. krusei* was the most prevalent species in ICUs (n=62, 45.92%). Figure 1 indicates the results for each step of molecular identification for randomly selected isolates. 

### Antifungal Susceptibility Testing:

Table 3 summarizes the MIC range, MIC50, MIC90, and GM MIC of eight antifungal drugs against identified different species. The assay was performed for strains isolated from each patient only once. In other words, the identical species isolated from each site at each time point were unified and repetitive species isolated from each patient were omitted. 

**Table 3:** The geometric mean, MIC50 and MIC90 values obtained by testing the susceptibility of isolated *Candida* species

Antifungal agent	*Candida* species (n)	MIC* range/MIC (µg/ml)	MIC50**(µg/ml)	MIC90€(µg/ml)	GM
Amphotericin	*C. albicans (13)*	0.125-4	0.5	2	0.6771
	*C. dubliniensis (3)*	0.25-1			0.5612
	*C. parapsilosis (4)*	0.5-2			0.6597
	*C. glabrata (1)*	0.5			0.5
	*C. krusei (16)*	0.5-2	0.5	2	0.7348
	*C. guilliermondii (2)*	0.125-0.5			0.3789
	*C. africana (1)*	0.25			1
	*C. lusitaniae (1)*	1			0.5
Fluconazole	*C. albicans*	0.125-16	1	4	0.6898
	*C. dubliniensis*	0.125-1			0.4851
	*C. parapsilosis*	1-2			0.7578
	*C. glabrata*	1			1
	*C. krusei*	0.5-16	1	8	0.8203
	*C. guilliermondii*	1-8			0.3077
	*C. africana*	0.125			0.125
	*C. lusitaniae*	0.5			0.5
Voriconazole	*C. albicans*	0.031-2	0.062	0.027	0.0777
	*C. dubliniensis*	0.031-0.125			0.0609
	*C. parapsilosis*	0.016			0.415
	*C. glabrata*	0.0125			0.0125
	*C. krusei*	0.016-0.25	0.062	0.25	0.0727
	*C. guilliermondii*	0.031-1			0.0474
	*C. africana*	0.016			0.016
	*C. lusitaniae*	0.031			0.031
Itraconazole	*C. albicans*	0.0625-16	0.25	0.5	0.2614
	*C. dubliniensis*	0.125-0.5			0.2024
	*C. parapsilosis*	0.125			0.1894
	*C. glabrata*	0.25			0.25
	*C. krusei*	0.125-16	0.25	0.5	0.2973
	*C. guilliermondii*	0.125-0.25			0.203
	*C. africana*	0.25			0.25
	*C. lusitaniae*	0.5			0.5
Ravuconazole	*C. albicans*	0.016-0.5	0.0625	0.237	0.515
	*C. dubliniensis*	0.016-0.0125			0.0574
	*C. parapsilosis*	0.0625-1			0.0950
	*C. glabrata*	0.031			0.031
	*C. krusei*	0.016-0.25	0.0625	0.162	0.0514
	*C. guilliermondii*	0.0625			0.625
	*C. africana*	0.25			0.25
	*C. lusitaniae*	0.0625			0.0625
Isavuconazole	*C. albicans*	0.016-1	0.031	0.0625	0.0364
	*C. dubliniensis*	0.016-0.0625			0.0331
	*C. parapsilosis*	0.031-0.0625			0.0356
	*C. glabrata*	0.016			0.016
	*C. krusei*	0.016-0.0125	0.031	0.0625	0.0328
	*C. guilliermondii*	0.016-0.031			0.0291
	*C. africana*	0.031			0.031
	*C. lusitaniae*	0.0625			0.625
Caspofungin	*C. albicans*	0.016-16	0.031	0.5	0.0537
	*C. dubliniensis*	0.016-0.0625			0.0354
	*C. parapsilosis*	0.016			0.016
	*C. glabrata*	0.016			0.016
	*C. krusei*	0.016-16	0.031	0.65	0.0582
	*C. guilliermondii*	0.031-0.125			0.0359
	*C. africana*	0.016			0.016
	*C. lusitaniae*	0.31			0.31
Anidulafungin	*C. albicans*	0.016-16	0.031	0.95	0.0629
	*C. dubliniensis*	0.031			0.031
	*C. parapsilosis*	0.031-0.125			0.0475
	*C. glabrata*	0.125			0.125
	*C. krusei*	0.016-4	0.031	0.65	0.0584
	*C. guilliermondii*	0.031-0.016			0.0239
	*C. africana*	0.016			0.016
	*C. lusitaniae*	0.016			0.016

* MIC: Minimum inhibitory concentration.

** MIC50: minimal concentration that inhibits 50 % of isolates.

€ MIC90: minimal concentration that inhibits 90 % of isolates.

¥ GM: Geometric mean. MIC50 /MIC90 were calculated only for the species with more than 5 strains in number.

Among 40 isolates, FLZ-resistant isolates (n=11, 8.15%) were determined according to the new breakpoints [ 26 ]. The strains include *C. albicans* (n=3/12), *C. krusei* (n=7/16), and *C. guillermondii* (n=1/1). According to extracellular volume values, two *C. dubliniensis* had high MICs against FLZ (0.5-1µg/ml). Among FLZ-resistant strains, one *C. albicans* and four *C. krusei* isolates were multi-azole resistant (3.7%). In the case of echinocandins, 4 isolates (2.96%) including *C. albicans* (n=1), *C. krusei* (n=1), and *C. parapsilosis* (n=1) were resistant against CAS and two isolates, including *C. albicans* (n=1) and *C. krusei* (n=1), were resistant against both CAS and AFG (totally 1.48%).

## Discussion

The first step for developing candidemia is colonization which has been evaluated in the present study. The most predominant species was reported as *C. krusei* in IICU, where the yeast hosts have weak immune systems and delicate skin/gut barriers. Hence, emphasizing the importance of species-level identification of Candida isolates is outstanding. However, according to our AFST results, only 7.2% of the strains were resistant to FLZ. 

Recent FLZ exposure may increase the potential for colonization and subsequent infection from FLZ-resistant *Candida* species [ 27 ]. Specifically, prior FLZ therapy, at any dose, has been associated with candidemia due to FLZ-resistant *Candida* isolates, including *C. glabrata*, *C. krusei*, and *C. tropicalis* [ 28 - 30 ]. Globally, C. krusei candidemia is an uncommon infection both in adults and pediatrics [ 2 ]; however, several outbreaks were reported due to the species in neonatal units [ 2 , 31 ]. Nevertheless, an unexpected increase was reported in the number of *C. krusei* candidemia cases from 4.93% in 2012 to 44.09% in 2014 [ 31 ]. 

Van Schalkwyk et al. reported a significant outbreak of candidemia with *C. krusei* as the causative agent among infants of Gauteng Province in South Africa from July to October 2014. Among 589 neonatal cases, 48 episodes of *C. krusei* candidemia occurred with an incidence of 8.2/100 admissions. In the aforementioned study, they identified 10 different species of Candida, and the most common one was *C. krusei* (35%) [ 31 ]. 

Kaur et al. reported a total of 316 cases of candidemia that were detected during 1 year in 2014. *Candida krusei* (30.3%) was the commonest isolate. The majority (58.8%) of cases were below 18 years of age and among the pediatric group, and 74.7% of them were neonates. *Candida krusei* candidemia significantly affected the pediatric group (44%), compared to adults (10.8%) [ 2 ]. The use of FLZ prophylaxis is considered a risk factor for the majority of *C. krusei* outbreaks [ 31 , 32 ] which was applied in the studied units for all patients. 

In Iran, Rezaei et al. conducted a study in Namazi NICU, Fars province, Iran. They screened 105 newborns in the NICU for fungal colonization, and the prevalence rate was 45.7%. In their study, fungal colonization was not associated with several factors, such as the gestational age, age, birth weight, gender, and duration of ICU admission of the newborns [ 33 ]. Hamzavi et al. performed another study during 2016–2017 on the children hospitalized in a referral oncology teaching hospital, in Shiraz, Iran. They found that nearly 60% of all the investigated children were colonized with different *Candida* species. Moreover, *C. krusei* was the most prevalent type of non-albicans species in colonized children [ 34 ]. 

Charsizadeh et al. reported the frequency, not colonization, of *Candida* species isolated from patients in a children’s medical center in 2017, the same hospital investigated in the present study. They found that *C. albicans* was the species most frequently isolated and detected in 77% of the patients. *Candida tropicalis* was the second-most common species (8.5%) identified in their study [ 35 ]. 

In the present study, FLZ prophylaxis was employed for all patients; hence, the application of FLZ as a prophylaxis antifungal might have promoted a considerable risk factor. It is well-known that more than 95% of clinical and veterinary isolates of *C. krusei* are intrinsically resistant to FLZ [ 36 , 37 ]. However, the mechanisms of resistance are not fully understood yet, while the efflux pump activity of the ATP-binding cassette transporter ABC1 as well as reduced FLZ affinity to Lanosterol 14 alpha-demethylase (encoded by *ERG11*) have been associated with this phenotypic feature [ 37 , 38 ]. Although the high distribution rate of *C. krusei* in PICU is a great concern, according to our AFST results, only 7.2% of our strains were resistant to FLZ. 

The main limitation of the present study may be the relatively small number of pediatric patients enrolled and the insufficiency of the duration of the study which limited us in following up on the patients.

## Conclusion

In the present study, since *C. krusei* is intrinsically resistant to FLZ, emphasizing the importance of species-level identification of *Candida* isolates is outstanding. However, according to our AFST results, only 7.2% of our strains were resistant to FLZ. Monitoring ICU patients who are at high risk of IC would be beneficial.

## Acknowledgments

The authors express their special thanks to the staff of the Children Medical Center in Tehran, Iran. This research was supported by Mazandaran University of Medical Sciences (Sari, Iran) .

## Authors’ contribution

M. M. conceived of the subjects of the study. AH. D. and J. J. performed the experiments. T. SH., MT. H., M. A., and B. N. advised the whole process of the study. All authors read and approved the final manuscript. 

## Conflicts of interest

There is no conflict of interest.

## Financial disclosure

The study was approved by the Ethics Committee of Mazandaran University of Medical Sciences under the code IR.MAZUMS.REC.1399.7165.

## References

[1] Caggiano G, Puntillo F, Coretti C, Giglio M, Alicino I, Manca F, et al ( 2011). Candida colonization index in patients admitted to an ICU. Int J Mol Sci.

[2] Kaur H, Shankarnarayana SA, Hallur V, Muralidharan J, Biswal M, Ghosh AK, et al ( 2020). Prolonged outbreak of Candida krusei candidemia in paediatric ward of tertiary care hospital. Mycopathologia.

[3] Kaur H, Chakrabarti A ( 2017). Strategies to reduce mortality in adult and neonatal candidemia in developing countries. J Fungi (Basel).

[4] Yapar N, Pullukcu H, Avkan-Oguz V, Sayin-Kutlu S, Ertugrul B, Sacar S, et al ( 2011). Evaluation of species distribution and risk factors of candidemia: a multicenter case-control study. Med Mycol.

[5] Pfaller MA, Moet GJ, Messer SA, Jones RN, Castanheira M ( 2011). Geographic variations in species distribution and echinocandin and azole antifungal resistance rates among Candida bloodstream infection isolates: report from the SENTRY Antimicrobial Surveillance Program (2008 to 2009). J Clin Microbiol.

[6] Steinbach WJ, Roilides E, Berman D, Hoffman JA, Groll AH, Bin-Hussain I, et al ( 2012). Results from a prospective, international, epidemiologic study of invasive candidiasis in children and neonates. Pediatr Infect Dis J.

[7] Messer SA, Jones RN, Fritsche TR ( 2006). International surveillance of Candida spp. and Aspergillus spp.: report from the SENTRY Antimicrobial Surveillance Program (2003). J Clin Microbiol.

[8] Clancy CJ, Yu VL, Morris AJ, Snydman DR, Nguyen MH ( 2005). Fluconazole MIC and the fluconazole dose/MIC ratio correlate with therapeutic response among patients with candidemia. Antimicrob Agents Chemother.

[9] Jarvis WR ( 1996). The epidemiology of colonization. Infect Control Hosp Epidemiol.

[10] Alenazy H, Alghamdi A, Pinto R, Daneman N ( 2021). Candida colonization as a predictor of invasive candidiasis in non-neutropenic ICU patients with sepsis: A systematic review and meta-analysis. Int J Infect Dis.

[11] León C, Ruiz-Santana S, Saavedra P, Almirante B, Nolla-Salas J, Álvarez-Lerma F, et al ( 2006). A bedside scoring system (“Candida score”) for early antifungal treatment in nonneutropenic critically ill patients with Candida colonization. Crit Care Med.

[12] León C, Ruiz-Santana S, Saavedra P, Galván B, Blanco A, Castro C, et al ( 2009). Usefulness of the “Candida score” for discriminating between Candida colonization and invasive candidiasis in non-neutropenic critically ill patients: a prospective multicenter study. Crit Care Med.

[13] Leroy O, Bailly S, Gangneux JP, Mira JP, Devos P, Dupont H, et al ( 2016). Systemic antifungal therapy for proven or suspected invasive candidiasis: the AmarCAND 2 study. Annals of intensive care.

[14] Slotman GJ, Shapiro E, Moffa SM ( 1994). Fungal sepsis: multisite colonization versus fungemia. Am Surg.

[15] Agvald-Öhman C, Klingspor L, Hjelmqvist H, Edlund C ( 2008). Invasive candidiasis in long-term patients at a multidisciplinary intensive care unit: Candida colonization index, risk factors, treatment and outcome. Scand J Infect Dis.

[16] Charles PE, Dalle F, Aube H, Doise JM, Quenot JP, Aho LS, et al ( 2005). Candida spp. colonization significance in critically ill medical patients: a prospective study. Intensive Care Med.

[17] Pfaller M, Neofytos D, Diekema D, Azie N, Meier-Kriesche HU, Quan SP, et al ( 2012). Epidemiology and outcomes of candidemia in 3648 patients: data from the Prospective Antifungal Therapy (PATH Alliance(R)) registry, 2004-2008. Diagn Microbiol Infect Dis.

[18] Altintop YA, Ergul AB, Koc AN, Atalay MA ( 2019). Evaluation of Candida colonization and use of the Candida Colonization Index in a paediatric Intensive Care Unit: a prospective observational study. Infez Med.

[19] Dimopoulos G, Ntziora F, Rachiotis G, Armaganidis A, Falagas ME ( 2008). Candida albicans versus non-albicans intensive care unit-acquired bloodstream infections: differences in risk factors and outcome. Anesth Analg.

[20] Gökahmetoğlu G, Sarıgüzel FM, Koc AN, Behret O, Gökahmetoğlu S, Atalay MA, et al ( 2016). Determination of Candida colonization and Candida score in patients in anesthesia intensive care unit. Mikrobiyol Bul.

[21] Vincent JL, Rello J, Marshall J, Silva E, Anzueto A, Martin CD, et al ( 2009). International study of the prevalence and outcomes of infection in intensive care units. JAMA.

[22] Eggimann P, Pittet D ( 2014). Candida colonization index and subsequent infection in critically ill surgical patients: 20 years later. Intensive Care Med.

[23] Romeo O, Criseo G ( 2008). First molecular method for discriminating between Candida africana, Candida albicans, and Candida dubliniensis by using hwp1 gene. Diagn Microbiol Infect Dis.

[24] Arastehfar A, Fang W, Pan W, Liao W, Yan L, Boekhout T ( 2018). Identification of nine cryptic species of Candida albicans, C. glabrata, and C. parapsilosis complexes using one-step multiplex PCR. BMC Infect Dis.

[25] CLSI (2012). Clinical and Laboratory Standards Institute. Reference method for broth dilution antifungal susceptibility testing of yeasts; fourth informational supplement. CLSI document M27-S4.

[26] CLSI ( 2008). Clinical and Laboratory Standards Institute. Reference method for broth dilution antifungal susceptibility testing of yeasts; fourth informational supplement. CLSI document M27-A2.

[27] Shah DN, Yau R, Lasco TM, Weston J, Salazar M, Palmer HR, et al ( 2012). Impact of prior inappropriate fluconazole dosing on isolation of fluconazole-nonsusceptible Candida species in hospitalized patients with candidemia. Antimicrob Agents Chemother.

[28] Garnacho-Montero J, Díaz-Martín A, García-Cabrera E, Ruiz Pérez de Pipaón M, Hernández-Caballero C, Aznar-Martín J, et al ( 2010). Risk factors for fluconazole-resistant candidemia. Antimicrob Agents Chemother.

[29] Lortholary O, Desnos-Ollivier M, Sitbon K, Fontanet A, Bretagne S, Dromer F ( 2011). Recent exposure to caspofungin or fluconazole influences the epidemiology of candidemia: a prospective multicenter study involving 2,441 patients. Antimicrob Agents Chemother.

[30] Slavin MA, Sorrell TC, Marriott D, Thursky KA, Nguyen Q, Ellis DH, et al ( 2010). Candidaemia in adult cancer patients: risks for fluconazole-resistant isolates and death. J Antimicrob Chemother.

[31] Van Schalkwyk E, Iyaloo S, Naicker SD, Maphanga TG, Mpembe RS, Zulu TG, et al ( 2018). Large outbreaks of fungal and bacterial bloodstream infections in a neonatal unit, South Africa, 2012–2016. Emerg Infect Dis.

[32] Patted SV, Halkati PC, Yavagal ST, Patil R ( 2009). Candida krusei infection presenting as a right ventricular mass in a two month old Infant. Ann Pediatr Cardiol.

[33] Rezaei M, Moghtaderi M, Badiee P, Zahadatpoor Z, Pooladfar G ( 2019). Fungal colonization among iranian infants hospitalized in the neonatal intensive care unit: occurrence rate, risk factors and health outcome. Int J Pediatr.

[34] Hamzavi S, Amanati A, Badiee P, Kadivar MR, Jafarian H, Ghasemi F, et al ( 2019). Changing face of Candida colonization pattern in pediatric patients with hematological malignancy during repeated hospitalizations, results of a prospective observational study (2016-2017) in shiraz, Iran. BMC Infect Dis.

[35] Charsizadeh A, Nikmanesh B, Ahmadi B, Jalalizand N, Jafari Z, Rahmani M, et al ( 2018). Frequency of Candida species isolated from patients in children’s Medical Center, Tehran, Iran. Arch Pediatr Infect Dis.

[36] Du J, Wang X, Luo H, Wang Y, Liu X, Zhou X ( 2018). Epidemiological investigation of non-albicans Candida species recovered from mycotic mastitis of cows in Yinchuan, Ningxia of China. BMC Vet Res.

[37] Whaley SG, Berkow EL, Rybak JM, Nishimoto AT, Barker KS, Rogers PD ( 2017). Azole antifungal resistance in Candida albicans and emerging non-albicans Candida species. Front Microbiol.

[38] Katiyar S, Edlind T ( 2001). Identification and expression of multidrug resistancerelated ABC transporter genes in Candida krusei. Med Mycol.

